# Systematic Review and Meta-Analysis of Randomized, Controlled Trials on Preoperative Physical Exercise Interventions in Patients with Non-Small-Cell Lung Cancer

**DOI:** 10.3390/cancers11070944

**Published:** 2019-07-05

**Authors:** Ilem D. Rosero, Robinson Ramírez-Vélez, Alejando Lucia, Nicolas Martínez-Velilla, Alejandro Santos-Lozano, Pedro L. Valenzuela, Idoia Morilla, Mikel Izquierdo

**Affiliations:** 1 Department of Health Sciences, Public University of Navarra, Navarrabiomed-Biomedical Research Centre, IDISNA-Navarra’s Health Research Institute, C/irunlarrea 3, Complejo Hospitalario de Navarra, 31008 Pamplona, Navarra, Spain; 2 Research Institute of the Hospital 12 de Octubre (i + 12), 28670 Madrid, Spain; 3 Faculty of Sport Sciences, Universidad Europea de Madrid, 28670 Madrid, Spain; 4 Centro de Investigación Biomédica en Red de Fragilidad y Envejecimiento Saludable (CIBERFES), Instituto de Salud Carlos III, 28029 Madrid, Spain; 5 i+HeALTH, Department of Health Sciences, European University Miguel de Cervantes, 47012 Valladolid, Spain; 6 Department of Systems Biology, University of Alcalá, 28805 Madrid, Spain

**Keywords:** resistance training, cardiovascular, functional capacity, lung cancer

## Abstract

Preoperative physical exercise protocols prior to cancer surgery increased in popularity over recent years; however, the beneficial effect of such protocols is not well established, with conflicting results reported. We conducted a systematic review and meta-analysis of randomized controlled trials (RCTs) to assess the effects of different modalities or combinations of preoperative exercise interventions and/or prehabilitation multicomponent training in patients with non-small-cell lung cancer (NSCLC) after surgery on the outcomes related to functional capacity, mental wellness and medical care. We searched in OVID Embase, Pubmed, Cochrane Library, CINAHL, Scopus, and Web of Science. Characteristics of studies and program results and outcome data were extracted. Changes between the intervention and control groups, from baseline to follow-up (standardized mean difference (SMD) or relative risk (RR) with 95% confidence interval (CI) for each intervention was pooled using weighted random-effects models). A total of 676 participants from 10 RCTs were included in the final analysis (aerobic training + inspiratory muscle training, *n* = 5; aerobic training + strength training + inspiratory muscle training, *n* = 2; aerobic training + strength training, *n* = 1; multicomponent training, *n* = 1; aerobic training alone, *n* = 1). The results showed intervention-induced improvement in walking endurance (SMD = 0.27; 95% CI, 0.11 to 0.44; *I*^2^ = 0.0%), peak exercise capacity (SMD = 0.78; 95% CI, 0.35 to 1.21; *I*^2^ = 76.7%), dyspnoea (SMD = −0.30; 95% CI, −0.51 to −0.10; *I*^2^ = 0.0%), risk of hospitalization (SMD = −0.58; 95% CI, −0.97 to −0.20; *I*^2^ = 70.7%), and postoperative pulmonary complications (relative risk (RR) = 0.50; 95% CI, 0.39 to 0.66; *I*^2^ = 0.0%). For the functional capacity and medical care parameters, preoperative combined aerobic, resistance, and inspiratory muscle training was shown to be effective if comprising one to four weeks, performing 1–3 sessions per week, with moderate intensity (50% for endurance capacity). Further studies with larger samples and higher methodological quality are needed to clarify the potential benefits of preoperative exercise training for patients with NSCLC.

## 1. Introduction

Lung cancer (LC) is the leading cause of cancer death in both sexes, and is responsible for more than one-quarter (27%) of all cancer deaths [1]. According to recent data, LC accounted for approximately 13% of new cancer cases and over one-quarter of all cancer-related deaths in the last year [2], with non-small-cell lung cancer (NSCLC) comprising 85% of all LC cases [3]. Treatment options for LC varies depending on the stage, the grade, and fuctional capacity of the patient; however, for those at the early stage of NSCLC, it normally includes surgery and chemotherapy/chemoradiotherapy (either in combination or in isolation). In advanced stages, treatments such as surgery, chemotherapy, immunotherapy, and radiotherapy can help increase life expectancy [4]. Nevertheless, although adjuvant treatments can also adversely affect the symptoms (severe treatment-related side effects are common [5]) and the physical performance of patients, as reported in other cancer populations [6,7]. In the American Society of Clinical Oncology guidelines [8], systemic therapy is mentioned as a standard treatment, but there is no cure for patients with stage IV NSCLC. Recent technical advances, however, facilitated the delivery of curative-intent radiation doses to some stage IV patients.

Recent years saw a growing interest in non-invasive interventions for cancer patients such as exercise training—either before or after surgery—with the goals of maximizing exercise performance capacity, promoting autonomy, increasing participation in daily living activities, improving health-related quality of life (HRQoL), and decreasing emotional issues. Exercise interventions may also help patients tolerate cancer treatments and can also reduce fatigue levels [9]. Indeed, exercise training during preoperative LC treatment was demonstrated to be safe and feasible. It is associated with significant improvements in walking endurance, peak exercise capacity, and some domains of HRQoL, as well as in the reduction of dyspnoea and fatigue [10,11]. Unfortunately, these studies are modest in sample size and show heterogeneity; therefore, they have limited generalizability across interventions.

The optimal design of preoperative exercise interventions, tailored to NSCLC patients, is yet to be established as there is still a need to define the most relevant exercise prescription. In this study, we conducted a systematic review and meta-analysis of randomized controlled trials (RCTs) to assess the different modalities or combinations of preoperative physical exercise interventions, such as inspiratory muscle training (IMT), aerobic training, strength/resistance training, and/or multicomponent training, on the outcomes of functional capacity, mental wellness, and medical care in patients with NSCLC after surgery.

## 2. Methods

This systematic review is in line with the Preferred Reporting Items for Systematic Reviews and Meta-Analyses (PRISMA) guidelines [12] and with the Cochrane Back Review Group [13]. 

### 2.1. Information Sources and Searches

A systematic review of RCTs published from 1970 to 2018 was carried out to update the existing knowledge on the influence of preoperative physical exercise in patients with NSCLC. A comprehensive computerized search of OVID Embase, Pubmed, Cochrane Library, CINAHL, Scopus, and Web of Science was conducted for human studies in adults older than 18 years. The search terms used included the following:

Search Pubmed (aged[Mesh] OR aged[title] OR aging[Mesh] OR elder*[title] OR elderly OR older*) AND (exercise[Mesh] OR “Exercise therapy” [Mesh] OR Exercise*[title] OR “physical training” OR “physical endurance” OR “exercise training” OR “physical activity” OR “physical fitness” OR “rehabilitation” OR “physical therapy modalities” OR “exercise therapy”) AND (“Lung Neoplasms”[Mesh] OR “lung carcinoma” OR “pulmonary carcinoma” [title] OR “lung neoplasm” OR “lung neoplasms” OR “pulmonary neoplasm” OR “pulmonary neoplasms” OR “pulmonary cancer” OR “lung cancer”[title] OR “lung tumor”[title] OR “lung tumour”[title] OR “lung metastases” OR “Non-Small-Cell Lung” AND “general surgery” OR “pulmonary surgical procedures” OR “thoracic surgical procedures” OR “surgical procedures, operative” OR “surg*” OR “operat*” OR “resection”) Sort by: Author Filters: Clinical Trial; Publication date from 1970/01/01 to 2018/02/13; Humans. In addition, reference lists were examined to detect studies that were potentially eligible for inclusion. Studies reported in languages other than English were not explored. Search Strategy are available in Table S1.

### 2.2. Study Selection

Two researchers (Mikel Izquiero and Nicolas Martínez-Velilla) independently carried out the search. The major criterium for including a study in the systematic review was an RCT that investigated the effects of different programs of preoperative physical exercise in NSCLC. Studies with patients with chronic obstructive pulmonary disease (COPD) were also included. After removal of duplicates, two independent reviewers (Robinson Ramírez-Vélez and Ilem D. Rosero) screened all potentially eligible articles using the titles and abstracts. These authors then applied the eligibility criteria, after obtaining the full texts, and generated a final list of included articles through consensus. Studies of specific comorbid conditions associated with participants having a diagnosis of secondary cancer, cardiovascular disease, or other significant medical, psychiatric, or neurological problems were excluded because these results were not believed to be generalizable to the NSCLC population.

### 2.3. Outcome Assessment

As primary results, we evaluated the effect of a preoperative physical exercise program on functional capacity (6-min walk distance (6MWD) test, in meters) measured as the change between the intervention and control groups, from baseline to follow-up (standardized mean difference (SMD) or relative risk (RR)).

Secondary end-points included change in peak of oxygen consumption (VO_2_ peak, mL/kg/min), forced expiratory volume in one second (FEV_1_, L), peak expiratory flow (PEF, L/min), forced vital capacity (FVC, L), diffusion capacity of the lung for carbon monoxide (DLCO, mL/min/mmHg), dyspnoea, and ratings of perceived exertion (RPE; numerical and/or Borg scale), mental wellness HRQoL, physical and emotional function (domains), medical care (postoperative hospitalization, days), and postoperative pulmonary complications (PPCs, among the most frequent were pneumonia and atelectasis). 

### 2.4. Data Extraction and Quality Assessment

Two authors (Robinson Ramírez-Vélez and Ilem D. Rosero) independently extracted data using a data extraction form, including baseline characteristics of the study design, participant characteristics, methods, quality, exercise protocol description (according to the American College of Sports Medicine (ACSM), 2011) [14], and outcomes of interest. For studies not reporting outcomes as a mean difference between baseline and end point measurements, outcomes were calculated using reported baseline and end-point data. Quality of the included studies was evaluated for risk of bias qualitatively using the Physiotherapy Evidence Database (PEDro; www.pedro.org.au) [15]. The items selected for use in the methodological assessment of the included RCTs were as follows: random allocation, concealed allocation, baseline comparability, blind subjects, blind therapists, blind assessors, adequate follow-up, intention-to-treat analysis, between-group comparisons, point estimates, and variability. Each item was classified as “yes” or “no”, with a total score of 10. Additionally, each study was evaluated with respect to training intervention (i.e., type of exercise, duration, frequency, and intensity) following the ACSM guidelines for exercise testing and prescription, 2006 [16]. Exercise interventions were considered adequate if all of the aforementioned requirements were met. Each requirement was answered with “yes” or “no” and then a total score was assigned, adding up the individual elements, which resulted in possible scores from 0 to 4.

### 2.5. Data Syntheses and Statistical Analyses

Individual patient-level data were not available for the studies in this analysis; thus, tabular data were used. The results of the quantitative meta-analysis of the outcomes (6MWD, dyspnoea, fatigue, the results of pulmonary function (FEV_1_, VO_2_peak, PEF, FVC, DLCO), HRQoL, physical and emotional function, postoperative hospitalization, and PPCs) were summarized using weighted random-effects (RE) models as SMD/RR statistics (with 95% confidence interval (CI)) at last follow-up between the experimental and control groups due to expected heterogeneity. Analyses of each intervention were also stratified by exercise regimen and interest outcomes. Heterogeneity was assessed among studies using the *I*^2^ statistic within each study group and within subgroups. *I*^2^ values of <25% and ≥50% were considered to be minimal and substantial, respectively. Publication bias was assessed with a visual inspection of funnel plots and with the Begg-Mazumdar Kendall’s tau and Egger bias tests. All analyses were carried out using R software, version 3.4.3 (R Foundation for Statistical. Computing, Vienna, Austria).

### 2.6. Role of the Funding Source

This study was funded by the Government of Navarra, Spain (grant no. 183/2018). The funder had no role in the study’s design, conduct, or reporting.

### 2.7. Ethics

An institutional research ethics board review was not required for this review of published literature.

## 3. Results

### 3.1. Characteristics of Included Trials and Participants

**Figure 1:**
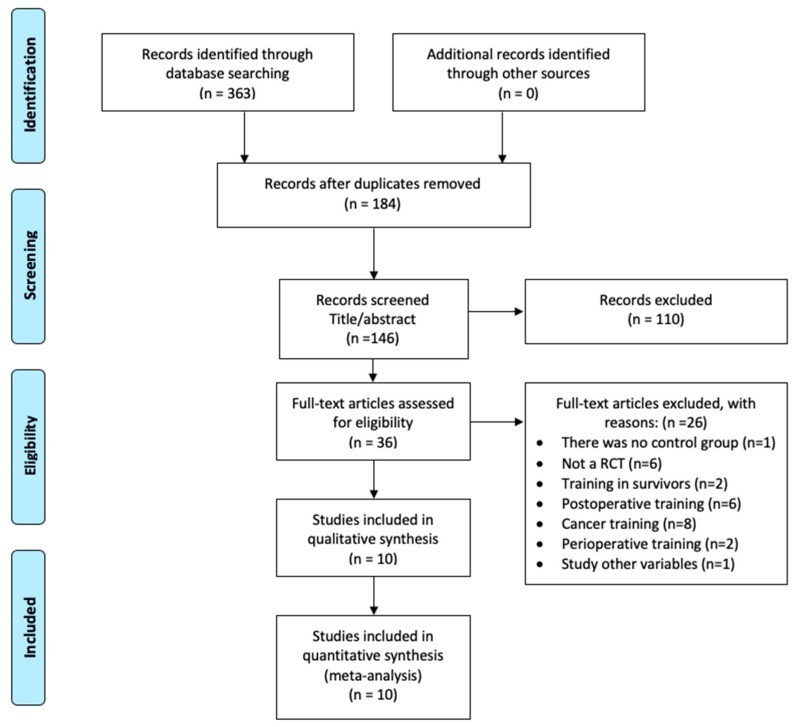
Preferred Reporting Items for Systematic Reviews and Meta-Analysis (PRISMA) flow diagram showing the number of studies identified and selected for inclusion in the systematic scoping review.

After a comprehensive literature search to identify relevant articles published between 1970 and 2018, 363 titles were initially screened for inclusion, 36 were assessed by full-text review, and 10 were included in the final meta-analysis (Figure 1). The studies included 676 participants (40.5% women), with stage I–IV NCSLC [17]. In addition, nine RCTs [18,19,20,21,22,23,24,25,26] included patients who were also diagnosed with COPD, and one trial [27] contained patients with a confirmed diagnosis of respiratory disease. All patients underwent surgery. The exercise (EG) and control (CG) groups did not differ with respect to preoperative patient characteristics, lung cancer stages (stage I–II: EG, 92%; CG, 93%; stage III–IV [19,20,21]: EG, 8%; CG, 7%), surgical procedures (open surgery: 51%; video-assisted thoracic surgery: 49%), and incidence of respiratory disease (COPD: EG, 54%; CG, 53%). Full details of other characteristics can be found in Table 1. 

### 3.2. Intervention Description

Concerning the exercise regimens for patients undergoing lung resection, supervised training was provided in specialized centers and lasted between one and four weeks, approximately. The intervention lasted one week in five studies [18,19,20,21,22], 2–4 weeks in four studies [23,24,25,26], and one study [27] did not specify the duration of the intervention but it lasted approximately four weeks (median 16 sessions). The duration of training sessions ranged between 20 and 60 min per session. Two studies performed two sessions per day [18,22], and the remaining studies performed three to seven sessions per week [19,20,21,23,24,25,26,27]. All studies included aerobic training (e.g., any type of endurance activity that increases the heart rate for a prolonged period (≥5 min) of time at 60–80% peak work capacity). Also, nine studies combined aerobic training with other training modalities: five studies [18,19,20,21,26] with inspiratory muscle training (IMT; abdominal and thoracic breathing exercises), two studies [22,27] with strength training and IMT, one study [24] with strength training, and one study [25] combined multicomponent training (aerobic training, strength training, and flexibility training including upper and lower limb stretching exercises) and IMT. Finally, we found one study [23] that included only aerobic training (Table 1). Exclusion criteria and the list of excluded articles are available in Table S2.

Regarding the practical applications and training prescription, we found five studies [18,19,20,21,22] in which exercise intensity was not reported, and one study [25] that did not report the duration of the exercise. Finally, four studies [23,24,26,27] were considered to have an adequate training intervention and a score of 4/4 (Table 2). All of the studies had a usual care group for comparison. In addition, one study [18] was three-armed and also compared the results with an IMT-only group.

### 3.3. Primary Outcome: Functional Capacity

**Figure 2:**
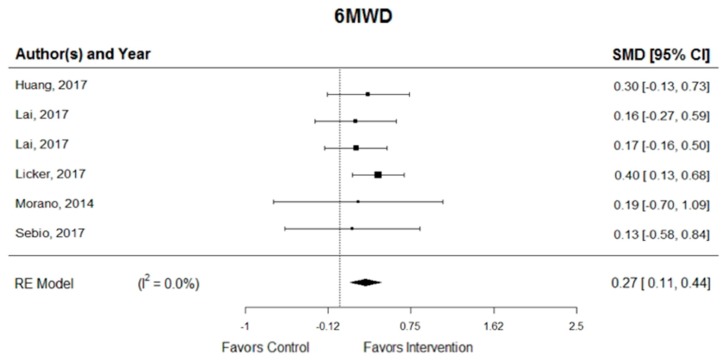
Pooled changes in functional capacity, six-minute walk distance (6MWD) by group. SMD = standardized mean difference; *I*^2^ = heterogeneity; RE = random-effects models.

In pooled analyses, the exercise intervention was associated with a significant improvement in 6MWD (SMD = 0.27; 95% CI, 0.11 to 0.44) [18,20,21,24,25,27] and one study showed a statistically significant effect (with the lower bound of the 95% CI > 0) [24]. In addition, homogeneity was substantial among the six RCTs for exercise intervention (*I*^2^ = 0.0%) (Figure 2). The symmetry of the funnel plot and a non-significant *p*-value in the Egger’s test together suggest that there was no significant publication bias (*p* = 0.970).

### 3.4. Secondary Outcomes 

**Figure 3:**
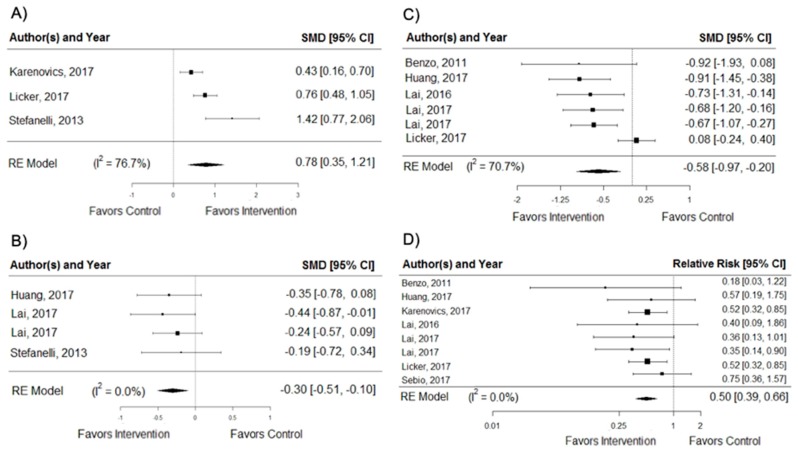
Pooled changes in secondary outcomes: (**A**) peak of oxygen consumption (VO_2_peak); (**B**) dyspnoea; (**C**) postoperative hospitalization (length of stay), and (**D**) postoperative pulmonary complications (PPCs). SMD = standardized mean difference; *I*^2^ = heterogeneity; RE = random-effects models.

Preoperatory exercise interventions resulted in a significant treatment change in VO_2_peak (SMD = 0.78; 95% CI, 0.35 to 1.21; *I*^2^ = 76.7%), dyspnoea (SMD = −0.30; 95% CI, −0.51 to −0.10; *I*^2^ = 0.0%), postoperative hospitalization (SMD = −0.58; 95% CI, −0.97 to −0.20; *I*² = 70.7%), and PPCs (RR = 0.50; 95% CI, 0.39 to 0.66; *I*^2^ = 0.0%), as shown in Figure 3. For the analysis of PPCs, two RCTs [18,20] used the complication classification system Clavien–Dindo, grade II to grade V, one RCT [27] used the Melbourne group scale, one trial [23] did not specify the classification, and the remaining studies [19,21,22,24] used their own parameters with the main pulmonary complications being pneumonia and atelectasis. The Egger test showed that there was no significant publication bias (VO_2_peak, *p* = 0.828; dyspnoea, *p* = 0.235; and PPCs, *p* = 0.928), except in length of stay (*p* = 0.008). There were no significant treatment effects or signs of publication bias on other secondary outcomes. Pooled changes in secondary outcomes are available in Figures S1 and S2. 

### 3.5. Quality Assessment

The quality of the included studies is shown in Table 3. Three studies did not specify eligibility criteria (30%) [21,24,26]. All of the studies presented an adequate random allocation. Six studies (60%) [18,21,23,24,25,27] reported allocation concealment. All the studies had groups similar at baseline, and they did not blind participants and therapists; however, six studies (60%) [18,20,21,23,24,27] reported assessor blinding. Three studies (30%) did not show adequate follow-up [19,26,27]. Five studies performed intention-to-treat analysis (50%) [18,20,23,24,25]. Finally, all the studies performed between-group comparisons, and showed point estimates and variability. 

### 3.6. Effect According to the Type of Intervention

#### 3.6.1. Combined Aerobic Exercise Training and IMT versus Usual Care

We found five RCTs [18,19,20,21,26] that combined aerobic training and IMT, and all analyzed their effects on dyspnea, but only one study (20%) [26] revealed significant differences between groups and time. In four of these studies (80%) [18,19,20,21], significant differences were found in the following outcomes: postoperative hospitalization, 6MWD, and PEF; in two of these studies [20,21], significant differences were found in PPCs, and only study [18] found significant differences in HRQoL. No significant differences were found in physical and emotional function in any of the trials. The remaining study (20%) [26] did not include these variables in the outcomes; however, significant differences in VO_2_peak and FEV_1_ were found.

#### 3.6.2. Combined Aerobic Exercise Training, Strength Training, and IMT versus Usual Care

Two RCTs were analyzed in this group [22,27], and both analyzed the effects on postoperative hospitalization, but no significant differences were found between groups. Only one study [22] reported significant differences in PPCs between groups. One study [27] showed significant differences in VO_2_peak and strength between groups and time from the first to the last evaluation, but did not find significant differences in 6MWD or physical function in HRQoL. Similar results were found by [25] using a multicomponent training regimen. This study showed significant differences in strength exercises and endurance testing in the period from the first to the last evaluation within the intervention group, as well as between groups. However, data did not show significant differences in 6MWD and HRQoL.

#### 3.6.3. Combined Aerobic Exercise Training and Strength Training versus Usual Care

One study [24] combined aerobic training + strength training and found significant differences between groups for 6MWD and VO_2_peak, as well as significant differences in the experimental group from baseline to post-intervention. In addition, significant differences were found between groups for the length of stay in the post-anesthesia care unit compared. However, no significant differences were found in the postoperative length in hospital or in PPCs.

#### 3.6.4. Aerobic Exercise Training versus Usual Care

Only one trial [23] reported significant changes for VO_2_peak and PPCs after aerobic exercise training. 

### 3.7. Effect According to the Duration and Frequency of the Intervention

#### 3.7.1. MWD

Change in 6MWD over the preoperative period was analyzed in six out of the 10 studies of the meta-analysis (60%) [18,20,21,24,25,27]. This variable showed significant results in four of these studies (67%). In three of them, the preoperative program was performed for one week, including two [18] or one sessions per day [20,21]. In the other study, the participants performed 2–3 sessions per week during 3–4 weeks [24]. Two out of the six studies (33%) did not show significant results [25,27]. Both studies performed an intervention of 3–5 sessions per week, one of them during for weeks [25] and the other did not specify the length of the intervention [27].

#### 3.7.2. VO_2_peak

This variable was analyzed in three out of 10 studies (30%) [23,24,26], showing significant results in all of them. In all of these interventions, the exercise program was carried out during a period of 2–4 weeks, performing 2–3 [23,24] or five sessions per week [26].

#### 3.7.3. Dyspnoea

This variable was analyzed in four studies (40%) [18,20,21,26], from which only one that lasted three weeks (five sessions per week) showed significant results [26]. The other three studies, which lasted one week, did not show significant results.

#### 3.7.4. Postoperative Hospitalization

This variable was analyzed in six studies (60%) [18,19,20,21,22,24]. Four of them (67%) showed significant results. They were all performed during one week, conducting one [19,20,21] or two sessions [18] per day. The other two studies (33%) did not show significant results. One of them performed a one-week intervention with two sessions per day [22], and the other one [24] consisted of 2–3 sessions per week during a period of 3–4 weeks.

#### 3.7.5. PPCs

This variable was the most widely analyzed (eight of 10 studies, 80%) [18,19,20,21,22,23,24,27]. Five of these studies (63%) showed significant results. Three out of these five studies performed the preoperative program for a week, once [20,21] or twice [22] a day, whereas the other two studies [23,24] performed the preoperative program 2–3 times a week during 2–4 weeks. Three out of the eight studies (37%), however, did not show significant results. Two of them lasted one week and included one [19] or two sessions [18] per day. The other study [27], which did not specify the duration (16 sessions), was performed 3–5 times per week.

## 4. Discussion

This systematic review aimed at determining the most effective preoperative physical exercise-based interventions (i.e., aerobic training, strength/resistance training, IMT, and/or multicomponent training) for patients treated surgically for NSCLC on outcomes of functional capacity, mental wellness, and medical care. According to the results of our systematic review, the literature in this field is lacking a sufficient number of RCTs. The pooled analyses suggest a significant beneficial effect on 6MWD, VO_2_peak, dyspnoea, postoperative hospitalization, and PPCs outcomes. Nevertheless, the findings of our study should be analyzed with caution because of the questionable methodological quality of the included studies. For instance, four studies did not describe how allocation was concealed, and, in four studies, assessors were not blinded. Furthermore, the intention-to-treat analysis was not reported/performed in five studies, which is likely to have affected the results. We also found the studies quite heterogeneous in terms of prescribed intervention, which makes it challenging to draw definitive conclusions according to the type of training.

Several studies confirmed that physical exercise is an effective treatment to improve exercise tolerance, reduce dyspnea, and improve quality of life in patients with cancer [10,22,26,28,29]. In a recent meta-analysis, Treanor and colleagues compared preoperative exercise with usual care in patients with cancer, reporting several benefits for LC patients in terms of pulmonary function, functional ability, health service utilization, and experience of treatment-related complications, even for patients with comorbidities [30]. In particular, the combination of aerobic exercise and IMT has a long history of research, especially in patients with poor lung function [31,32,33]. Indeed, there appears to be a potentially significant advantage in implementing preoperative exercise regimens rather than usual rehabilitation interventions for cancer patients [30]. 

Despite the heterogeneity between the studies analyzed here with respect to the intervention, we observed that all studies included aerobic exercise, which is considered the best way to improve VO_2_peak in healthy subjects [34], and it was also successfully prescribed to individuals with several chronic diseases [35,36,37]. We found beneficial effects on functional capacity and improvements in VO_2_peak after training. The 6MWD was one of the most common measures among the studies examined (6/10; 60%), and one of these six studies [24] reported an increase of more than 42 m, which was recently established as the upper limit of the minimally important difference in individuals with LC [38]. Another three studies [18,20,21] showed an increase in the distance walked, albeit not reaching this limit, and two studies [25,27] showed a decrease in distance in relation to the initial evaluation; however, the results were better when compared with the control group. 

VO_2_peak is the gold standard for evaluating cardiorespiratory fitness in healthy subjects [39,40], and is a strong and reliable predictor of postoperative mortality and morbidity, HRQoL, and long-term survival in NSCLC [39,41,42,43,44,45]. However, only three of the 10 studies (30%) [23,24,26] included this variable in their analysis, and an improvement after training was found in all of them.

Preoperative exercise was found to be effective in reducing postoperative complications and length of hospital stay in patients undergoing LC surgery, which is in agreement with a recent review [46]. According to [47], a reduction in the duration of hospital stay after exercise training may be associated with increased exercise capacity, muscle strength, and pulmonary function, and reduced fatigue. Therefore, physical exercise should be considered as a standard of preoperative care. These findings may impact the cost of medical care, which would be beneficial both for patients and for public healthcare systems in general, since postoperative morbidity is regarded as the main cause for increased overall hospital costs and long-term impairment [27]. 

PPCs were also shown to have an impact on cancer-related survival [48], increasing the risk of mortality irrespective of the stage of the disease [29]. In this meta-analysis, a significant reduction in PPCs was observed in the eight studies that analyzed this variable [18,19,20,21,22,23,24,27]. Regarding PPCs, only one of six studies [24] analyzing this variable reported a longer hospital stay in the exercise group in relation to the control group. Regarding dyspnoea, our findings are in line with previous research reporting that exercise training improves dyspnoea in postoperative patients [10,47], in particular when aerobic training is part of a preoperative pulmonary rehabilitation program in patients with a variety of chronic respiratory diseases [49,50,51,52]. A reduction in the perception of dyspnoea was found in four studies [18,20,21,26] that analyzed this variable. 

## 5. Limitations of the Review

The main limitation of this meta-analysis was the heterogeneity of the included trials. Chief amongst these was the variation in the methodological aspects between the RCTs, which introduced a moderate risk of bias; for example, some trials did not blind the research subjects, the intervention, and/or the outcome assessors. Other limitations included the rather broad range of intervention times between studies (approximately 1–4 weeks), the insufficient sample sizes in some studies, which implies a potential risk of overestimating positive results, and the lack of information in some studies about the prescription of the exercise (i.e., type, intensity, frequency, duration of training), which makes it difficult to interpret the results and especially the different variables analyzed in the studies. In this line, the main target cancer population for preoperative physical exercise interventions regimens is also yet to be established. However, supervised exercise interventions may be particularly beneficial for cancer patients who experienced muscle loss as a key component of cachexia or neoadjuvant therapy.

## 6. Conclusions and Future Recommendations

In the 10 RCTs analyzed, significant positive results were found in functional capacity (6MWD, VO_2_peak, dyspnoea) and medical care (preoperative hospitalization and PPCs). Regarding mental wellness, there were improvements and a tendency toward the intervention group demonstrating better outcomes, but the results were not statistically significant. Overall, we found that the implementation of a program of preoperative physical exercise in patients with NSCLC produces benefits and positive effects in the three domains. Nevertheless, it was difficult to draw robust conclusions on the best type of preoperative physical exercise for patients awaiting thoracic surgery. Studies with a duration as short as one week displayed a significant improvement in 6MWD, as well as a high probability of decreasing the length of postoperative hospitalization stay, which may involve a relevant decrease of morbidity and postoperative costs. However, in these particular conditions, a longer program (2–4 weeks) might be required in order to observe significant differences in VO_2_peak and dyspnoea.

Standardization of the methodological aspects of future RCTs is recommended to clarify the potential benefits of preoperative physical training in patients with NSCLC. Future studies should clearly describe the content of the exercise intervention, as well as the adherence rates to these interventions, and they should also report potential adverse events associated with the exercise sessions [53]. Further trials of higher quality and ideally involving several centers are required to confirm the effectiveness of training with preoperative exercises in patients with NSCLC.

## Figures and Tables

**Table 1:** Characteristics of included studies.

Reference, Year	Intervention	Duration/Frequency	*N*	Setting	Follow-Up Time (Days)	Participants	Outcomes of Interest	Feasibility/AE	Additional Notes	Main Findings
**Aerobic exercise training and inspiratory muscle training**										
Huang et al., 2017 [18]	Three conditions: 1. (a) IMT-ABT: two to three times daily for 15–20 min/s; -TBT (Voldyne 5000): 20 min at least four times daily; (b) CRT (NuStep): twice daily for 20 min/s; (c) psychological educational guidance (EG) 2. Single IMT (SG) 3. Routine preoperative preparation (CG)	1 week 2 times a day	60	In hospital	No follow-up	90 patients were randomized: age, mean (SD); Control group: *n* = 30, 63.6 (6.5); Exercise group: *n* = 30, 63.0 (8.7); Single group: *n* = 30, 64.1 (5.3)	Hospitalization postoperative days ^a^ PPCs ^c^ 6MWD (meters) ^ab^ PEF (L/min) ^ab^ FEV1 (L) ^cd^ FVC (L) ^cd^ Fatigue ^cd^ Dyspnoea ^cd^ DLCO (mL/min/mmHg) ^cd^ HRQoL ^ab^ Physical function ^cd^ Emotional function ^cd^	The completion rates suggest that compliance with the programs is likely to be high and also supports the feasibility of the EG program	Proportion of subjects who completed the pogram (97%). Adherence to the prescribed training sessions NR. SG did not show significant differences in outcomes compared with CG	This hospital-based short-term pattern of PPR combining high-intensity IMT with CRT could be a feasible strategy for treating NSCLC patients, especially those with risk factors of PPCs awaiting surgery
Lai et al., 2016 [19]	Two conditions: 1. I. Pharmacotherapy (Bricanyl, Pulmicort, Mucosolvan): 2 times/day; II. Physical rehabilitation: (a) IMT-ABT: (20–30 rep in 15–30 min), -TBT (Voldyne 5000): 2–3 s, 12–20 rep/2 h; (b) -EET-LE (NuStep): 15–20 min/day, -Climbing ladder training (EG): 15–30 min/day 2. Underwent standard preoperative (CG)	1 week Daily	48	In hospital	No follow-up	48 patients were randomized: age, mean (SD); Exercise group: *n* = 24, 63.13 (6.26); Control group: *n* = 24, 64.04 (8.94)	Hospitalization postoperative days ^a^ PEF (L/min) ^b^ 6MWD (meters) ^b^ HRQoL ^d^ Physical function ^d^ Emotional function ^d^ Dyspnoea ^d^ Fatigue ^d^ PPCs ^c^	No AE related to the intervention	Proportion of subjects who completed the pogram (100%). Adherence to the prescribed training sessions NR	Pre-operative short-term comprehensive pulmonary rehabilitation training can improve pulmonary resistance of patients with mild to moderate COPD, accelerate rapid recovery of patients after surgery, can be used as an important part of the PPR fast
Lai, Huang, et al., 2017 [20]	Two conditions: 1. (a) IMT-ABT: twice per day 15–20 min/s; -TBT (Voldyne 5000): 3 s/day 20 min/s. (b) EET (NuStep): 30 min/day 2. Usual care (CG)	1 week Daily	127	In hospital	No follow-up	60 patients were randomized: age, mean (SD); Exercise group: *n* = 30, 72.5 ± (3.4); Control group: *n* = 30, 71.6 ± (1.9)	Hospitalization postoperative days ^a^ PEF (L/min) ^ab^ FVC (L) ^c^ FEV1 (L) ^c^ DLCO (ml/min/mmHg) ^c^ 6MWD (meters) ^ab^ HRQoL ^c^ Physical function ^c^ Emotional function ^c^ Dyspnoea ^c^ PPCs ^a^	4 patients in the EG suspended the training because they could not endure the highly intensive regimen, 1 perceived a lack of benefit, and 1 suffered from knee pain	Proportion of subjects who completed the pogram (47%). Adherence to the prescribed training sessions NR	PPR played a positive physical role in improving the PEF and 6MWD in elderly surgical patients with LC while significantly reducing the postoperative length of stay. We, thus, consider the 7-day intensive pattern of PPR to be a feasible rehabilitation strategy for elderly NSCLC patients in China
Lai, Su, et al., 2017 [21]	Two conditions: 1. (a) IMT-TBT (HUDSON RCI 2500): 3 s/day, 20 rep/s; -ABT: twice per day 15–30 min/s. (b) EET (NuStep): 30 min/day 2. Usual care (CG)	1 week Daily	101	In hospital	No follow-up	101 patients were randomized: age, mean (SD); Exercise group: *n* = 51, 63.8 ± (8.2); Control group: *n* = 50, 64.6 ± (6.6) Range: (50–80 years)	Hospitalization postoperative days ^a^ PEF (L/min) ^ab^ 6MWD (meters) ^ab^ HRQoL ^cd^ Physical function ^cd^ Emotional function ^cd^ Dyspnoea ^cd^ Fatigue ^cd^ PPCs ^a^	No AE related to the intervention	Proportion of subjects who completed the pogram (94%). Adherence to the prescribed training sessions NR	Fewer PPCs and better postoperative recovery in the EG, which led to shorter postoperative hospital stays, reduced use of medications and medical care, consequently, lower in-hospital expenses
Stefanelli et al., 2013 [26]	Two conditions: 1. (a) IMT: Respiratory exercises on the bench, mattress pad and wall bar. (b) EET: High intensity training of UE (rowing ergometer) and LE (treadmill and the ergometric bicycle), 70% PWC (c) Pharmacotherapy (bronchodilators, inhaled corticosteroids) 2. Usual care (CG)	3 weeks 5 times a week	40	In hospital	60	40 patients were randomized: age, mean (SD); Exercise group: 65.5 (± 7.4) Control group: 64.8 (± 7.3)	FEV1 (L) ^ab^ VO_2_peak (mL/kg/min) ^ab^ DLCO (ml/min/mmHg) ^cd^ Dyspnoea ^ab^	NR	Proportion of subjects who completed the pogram NR. Adherence to the prescribed training sessions NR	It is possible to state that preoperative high-intensity PRP improves the degree of physical performance of patients with COPD and NSCLC undergoing surgical resection compared with similar surgical patients who did not undergo preoperative PRP
**Aerobic exercise training, strength training, and IMT**										
Benzo et al., 2011 [22]	Two studies: I. NR II. Two conditions: 1. (a) EET (LE/UE) (NuStep): 20 min (b) Strength alternating UE/LE every other day (Thera-band): 2s × 10–12 rep (c) (IMT) (Threshold IMT or the P-Flex valve) (EG): 15–20 min of daily use. 2. Usual care (CG)	I. 4 weeks II. 1 week 2 times a day	I. 9; II. 19	I. In hospital II. In hospital	No follow-up	I. 9 patients were randomized: EG *n* = 5; CG *n* = 4 II. 17 patients were randomized: age, mean (SD); Exercise group: *n* = 9, 70.2 (8.61); Control group: *n* = 8, 72.0 (6.69)	II. Hospitalization postoperative days ^c^ PPCs ^a^	I. Non-feasibility of 4 weeks of PPR. No AE related to the intervention	II. Proportion of subjects who completed the pogram (89%). Adherence to the prescribed training sessions NR	I. PPR is appropriate and recommended by experts. II. The development of a short and feasible PPR protocol was the natural consequence of the failure of the longer one. The 10-session protocol showed a high likelihood of decreasing hospital length of stay, a very meaningful outcome that is a crude estimation of postoperative morbidity and costs
Sebio García et al., 2017 [27]	Two conditions: 1. (a) EET moderate (cycle ergometer-Monark): -30 min interval training -5 min warm-up (30% PWC) -1 min (80% PWC) -4 min (active rest 50% PWC). -4 min cool down (30% PWC) (b) CRT: - elastic bands (Thera-Band^®^) body-weight exercises: six different exercises: 15 rep × 3 s, 45 s micropause (increased to 4 s if tolerated) (c) IMT (Coach 2 Incentive Spirometer^®^). -TBT: 2 s/day 30 sustained inspirations (80% MVC) end inspiratory hold (2–3 s). 6 cycles × 5 rep, 1 min pause/ cycle. 2. Usual care (CG)	Median of 16 sessions 3–5 times a week	40	In hospital	55	22 patients were randomized: age, mean (SD); Exercise group: *n* = 10, 70.9 ± (6.1); Control group: *n* = 12, 69.4 ± (9.4)	Hospitalization postoperative days ^c^ PPCs^c^ 6MWD (meters) ^cd^ Physical Component ^ad^ Physical functioning ^d^ Emotional function ^d^	No AE related to the intervention.	Proportion of subjects who completed the pogram (55%). Adherence to the prescribed training sessions NR	Although no significant differences between groups were observed at three weeks in any of the variables analyzed, three months postoperatively, there were statistically significant differences in the mean change for the exercise capacity, the physical component summary, and the upper and lower muscle strength assessment leading to two opposite trends in patients’ recovery
**Aerobic exercise training and strength training**										
Licker et al., 2017 [24]	Two conditions: 1. (a) Warm-up: 5 min (50% PWC); 2 s × 10 min [(interv 15 s (80–100% PWC), micropause 15 s, macropause 4 min/s; Cooled down: 5 min (30% PWC) (b) EET (cycle ergometer) (c) strengthening UE/LE: leg press, leg extension, back extension, seat row, biceps curls or chest and shoulder press 2. Usual care (CG)	3–4 weeks 2–3 times a week	164	In hospital	No follow-up	151 patients were randomized: age, mean (SD); Exercise group: *n* = 74, 64 (13); Control group: *n* = 77, 64 (10)	VO_2_peak (mL/kg/min) ^ab^ 6MWD (meters) ^ab^ PPCs ^a^ Hospitalization postoperative (days) ^c^	No AE related to the intervention	Proportion of subjects who completed the pogram (92%). Adherence to the prescribed training sessions. EG: 87 ± 18%	Demonstrated the safety and effectiveness of a short-term exercise training program in improving aerobic performances in patients LC. However, this HIIT rehabilitation modality failed to produce significant difference in composite morbidity-mortality index, compared with usual care
**Multicomponent training and IMT**										
Morano et al., 2014 [25]	Two conditions: 1. (a) Stretching LE/UE; (b) warm-up exercises; (c) strengthening UE (50% PWC), PNF (barbells); (d) EET (treadmill) 80% PWC; (e) IMT; (f) educational sessions 2. (a) CPT (routine protocol of the hospital comprising lung expansion techniques) -sustained maximum inspiration; -fractional inspiration; -breathing patterns; -pursed lip breathing; -use of a flow-based incentive spirometer (Respiron) (b) Educational sessions (CG)	4 weeks 5 times a week	31	In hospital	30	24 patients were randomized: age, mean (SD); Exercise group: *n* = 12, 65 ± (8); Control group: *n* = 12, 69 ± (7)	6MWD (meters) ^cd^ HRQoL ^cd^	No AE related to the intervention	Proportion of subjects who completed the pogram (100%). Adherence to the prescribed training sessions NR	The study showed improvements in PEF and 6MWD and reductions in the total / postoperative length of stay, hospital costs and occurrence of PPCs
**Only aerobic exercise training**										
Karenovics et al., 2017 [23]	Two conditions: 1. (a) Warm-up: 5 min (50% PWC); 2 s × 10 min (interv 15 s sprint and 15 s pause, macropause 4 min/s); Cooled down: 5 min (30% PWC) (b) EET (cycle ergometer) 2. Usual care (CG)	2–4 weeks 3 times a week	164	In hospital	30	151 patients were randomized: age, mean (SD); Exercise group: *n* = 74, 64 (13); Control group: *n* = 77, 64 (10)	VO_2_peak (mL/kg/min) ^a^ PPCs ^a^	No AE related to the intervention	Proportion of subjects who completed the pogram (92%). Adherence to the prescribed training sessions was 87 ± 18% (median 8 sessions, IQ 25–75% [7,8,9,10]) in EG	A HIIT program limited to the preoperative period is not associated with better functional and clinical outcome 1 year after lung cancer surgery

Preoperative pulmonary rehabilitation (PPR); lower extremity (LE); upper extremity (UE); experimental group (EG); control group (CG); single group (SG); exercise endurance training (EET); inspiratory muscle training (IMT); postoperative pulmonary complications (PPCs); adverse events (AE); standard deviation (SD); not reported (NR); abdominal breathing training (ABT); thoracic breathing training (TBT); 6-min walking distance (6MWD); peak expiratory flow (PEF); health-related quality of life (HRQoL); chest physical therapy (CPT); peak work capacity (PWC); unsupported upper limb exercise test (UULEX); whole-body vibration training (WBVT); conventional resistance training (CRT); maximal vital capacity (MVC); video-assisted thoracoscopy (VATS); non-small-cell lung cancer (NSCLC); forced vital capacity (FVC); peak of oxygen consumption (VO_2_peak); forced expiratory volume in one second (FEV_1_); diffusion capacity of the lung to carbon monoxide (DLCO); chronic obstructive pulmonary disease (COPD); high intensity interval training (HIIT); proprioceptive neuromuscular facilitation (PNF). ^a^ Significantly greater improvement the intervention compared with control; ^b^ significant program effects for exercise group from baseline to post intervention; no significant effect for controls; ^c^ no significant intervention difference between exercise and control groups; ^d^ without significant effects for the exercise group from the beginning to the post intervention.

**Table 2:** Analysis of preoperatory exercise training interventions.

Reference, Year	Type of Exercise	Duration	Frequency	Intensity	Total Score
Benzo et al., 2011 [22]	Yes	Yes	Yes	No	3/4
Huang et al., 2017 [18]	Yes	Yes	Yes	No	3/4
Karenovics et al., 2017 [23]	Yes	Yes	Yes	Yes	4/4
Lai et al., 2016 [19]	Yes	Yes	Yes	No	3/4
Lai, Huang, et al., 2017 [20]	Yes	Yes	Yes	No	3/4
Lai, Su, et al., 2017 [21]	Yes	Yes	Yes	No	3/4
Licker et al., 2017 [24]	Yes	Yes	Yes	Yes	4/4
Morano et al., 2014 [25]	Yes	No	Yes	Yes	3/4
Sebio García et al., 2017 [27]	Yes	Yes	Yes	Yes	4/4
Stefanelli et al., 2013 [26]	Yes	Yes	Yes	Yes	4/4

**Table 3:** Quality of the studies included in the meta-analysis (Physiotherapy Evidence Database, PEDro scale).

Study	Eligibility Criteria Specified *	Random Allocation	Concealed Allocation	Groups Similar at Baseline	Participant Blinding	Therapist Blinding	Assessor Blinding	Adequate Follow-Up	Intention-To-Treat Analysis	Between-Group Comparison	Point Estimates and Variability	Total Score
Benzo et al., 2011 [22]	Yes	Yes	No	Yes	No	No	No	Yes	No	Yes	Yes	6/10
Huang et al., 2017 [18]	Yes	Yes	Yes	Yes	No	No	Yes	Yes	Yes	Yes	Yes	8/10
Karenovics et al., 2017 [23]	Yes	Yes	Yes	Yes	No	No	Yes	Yes	Yes	Yes	Yes	8/10
Lai et al., 2016 [19]	Yes	Yes	No	Yes	No	No	No	No	No	Yes	Yes	4/10
Lai, Huang, et al., 2017 [20]	Yes	Yes	No	Yes	No	No	Yes	Yes	Yes	Yes	Yes	7/10
Lai, Su, et al., 2017 [21]	No	Yes	Yes	Yes	No	No	Yes	Yes	No	Yes	Yes	7/10
Licker et al., 2017 [24]	No	Yes	Yes	Yes	No	No	Yes	Yes	Yes	Yes	Yes	8/10
Morano et al., 2014 [25]	Yes	Yes	Yes	Yes	No	No	No	Yes	Yes	Yes	Yes	7/10
Sebio García et al., 2017 [27]	Yes	Yes	Yes	Yes	No	No	Yes	No	No	Yes	Yes	6/10
Stefanelli et al., 2013 [26]	No	Yes	No	Yes	No	No	No	No	No	Yes	Yes	4/10

* Eligibility criteria item does not contribute to total score.

## Supplementary Materials

The following are available online at https://www.mdpi.com/2072-6694/11/7/944/s1, Figure S1: Pooled changes in secondary outcomes: DLCO (Diffusion capacity of the lung for carbon monoxide); FVC (Forced vital capacity); FEV_1_ (Forced expiratory volume in one second); Fatigue and PEF (Peak expiratory flow). Figure S2: Pooled changes in secondary outcomes: Emotional function; Physical function; and Health-related quality of life. Table S1: Search Strategy, Table S2: Exclusion criteria and the list of excluded articles.
