# Supplementary material for: Systematic Review and Meta-Analysis of Randomized, Controlled Trials on Preoperative Physical Exercise Interventions in Patients with Non-Small-Cell Lung Cancer
Source: Cancers (Basel). 2019 Jul 5;11(7):944. doi: 10.3390/cancers11070944 (PMC6678369; doi:10.3390/cancers11070944)
Supplement: Supplementary file 1 [file cancers-11-00944-s001.pdf]

Supplementary Materials: Systematic Review and Meta-Analysis of Randomized, Controlled Trials on Preoperative Physical Exercise Interventions in Patients with Non-Small-Cell Lung Cancer

Ilem D. Rosero, Robinson Ramírez-Vélez, Alejandro Lucia, Nicolas Martínez-Velilla, Alejandro Santos-Lozano, Pedro L. Valenzuela, Idoia Morilla and Mikel Izquierdo

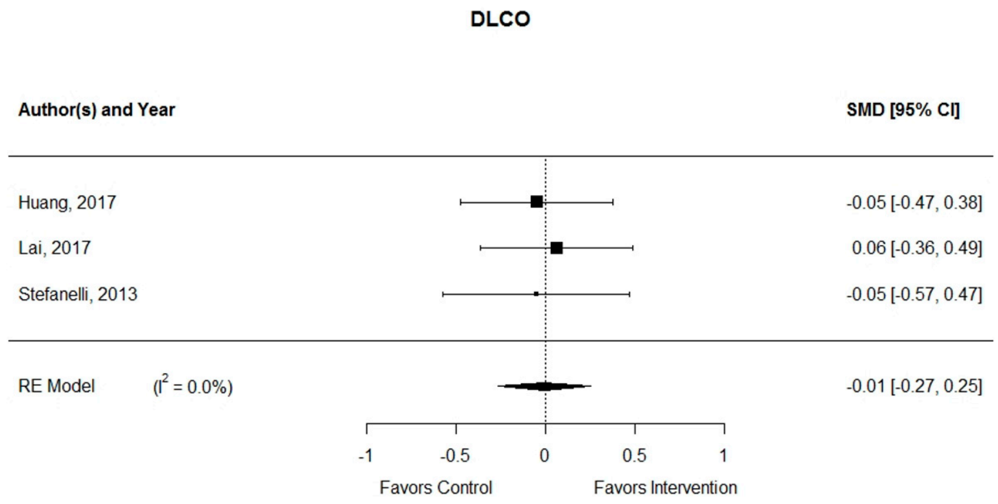

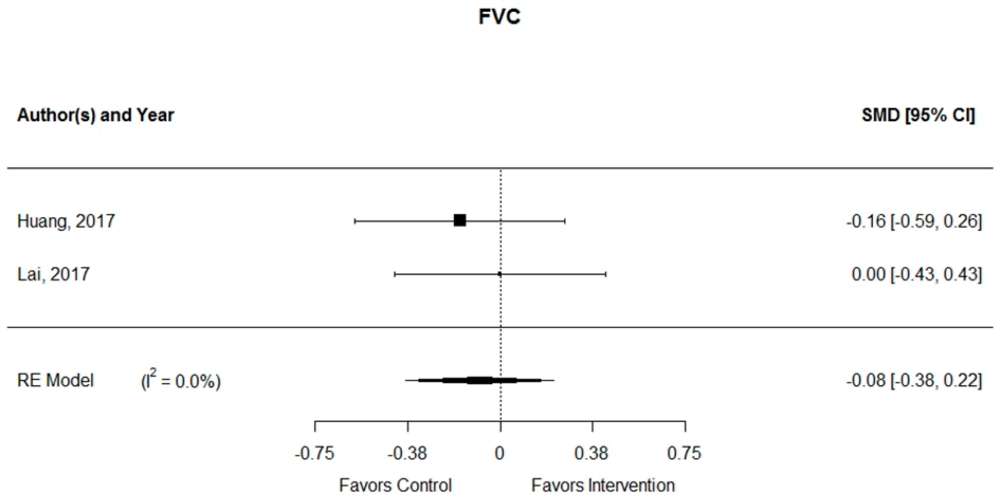

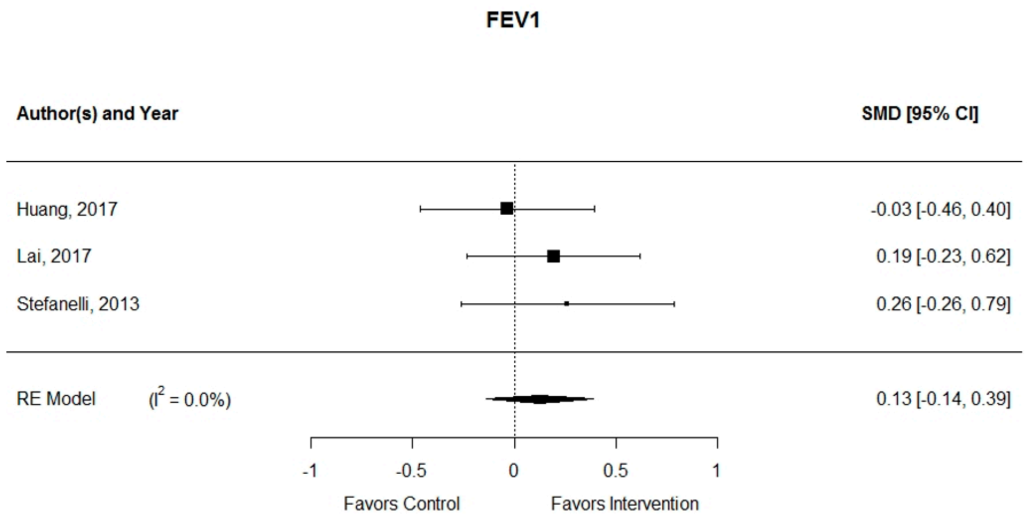

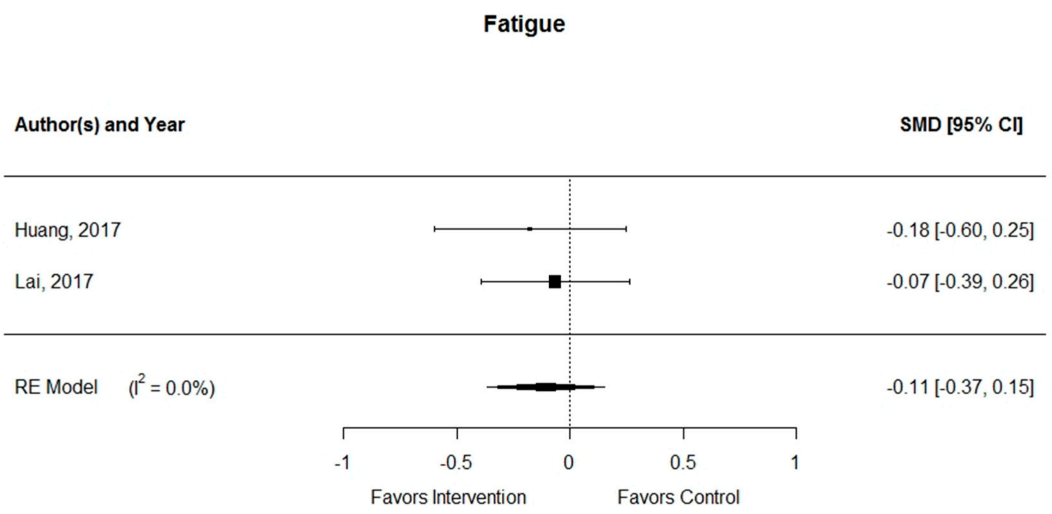

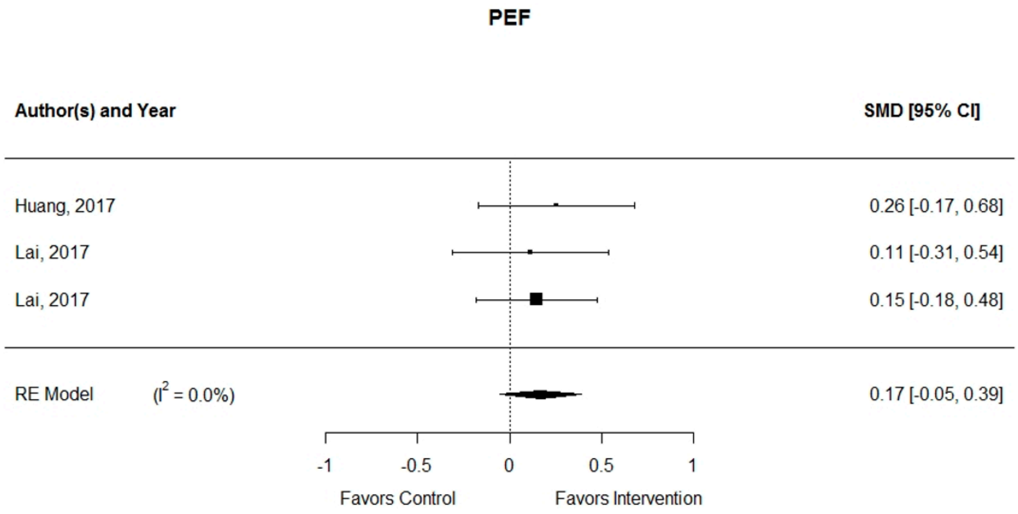

Figure S1. Pooled changes in secondary outcomes: DLCO (Diffusion capacity of the lung for carbon monoxide); FVC (Forced vital capacity); FEV_1 (Forced expiratory volume in one second); Fatigue and PEF (Peak expiratory flow).

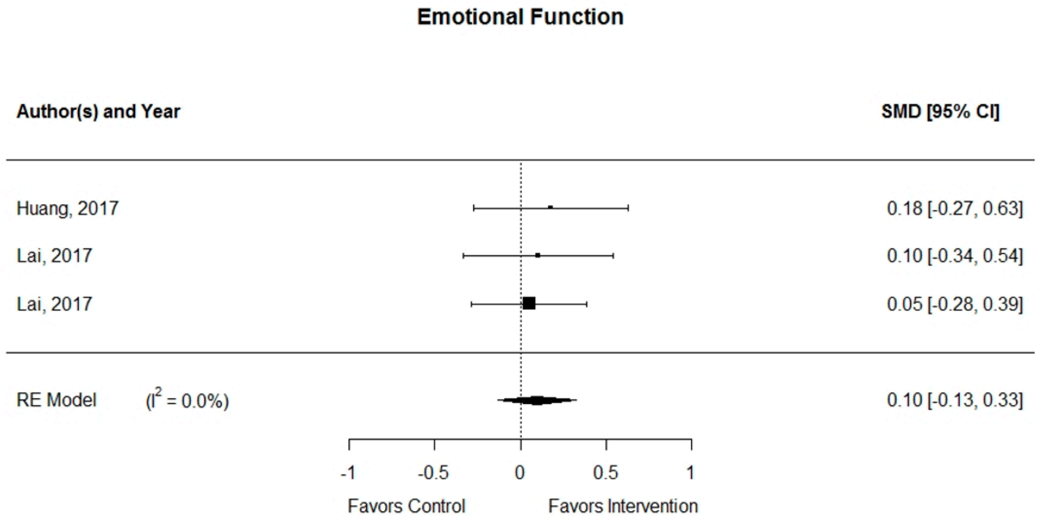

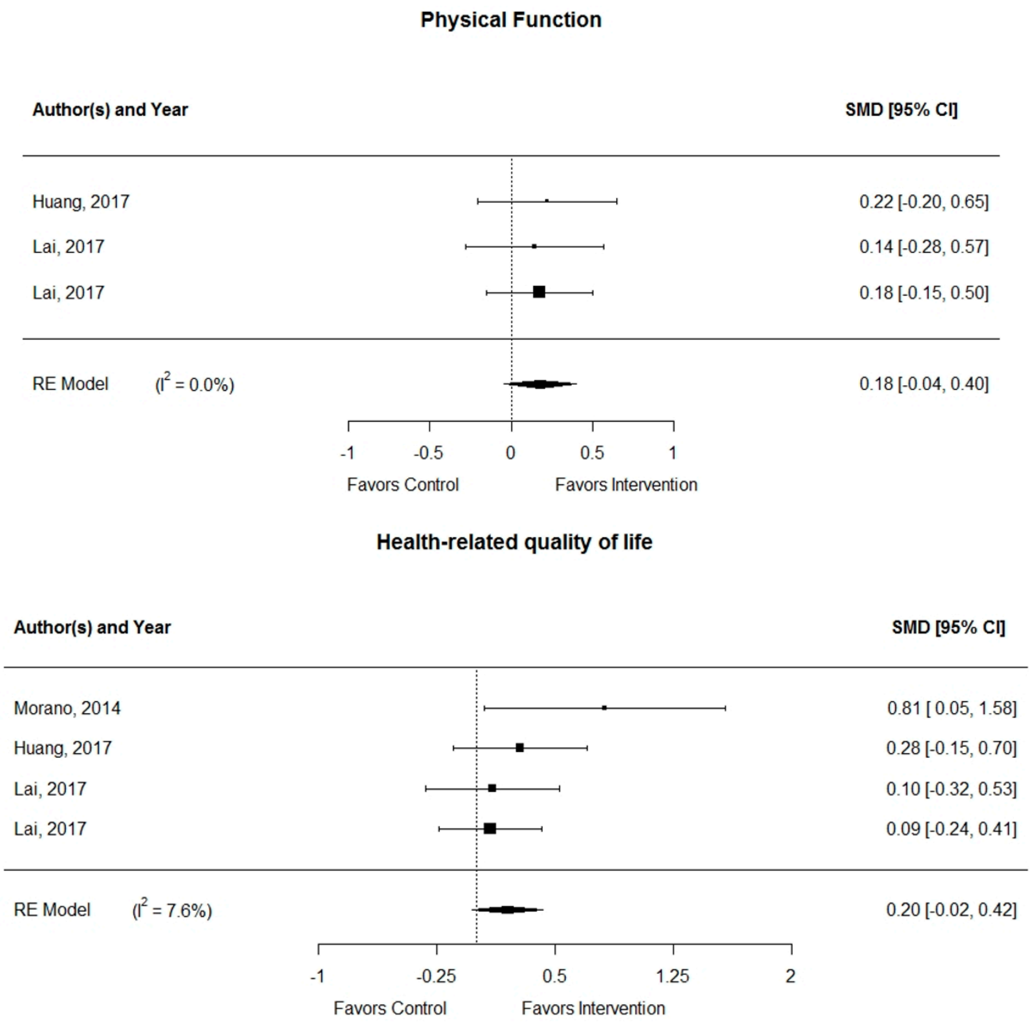

Figure S2. Pooled changes in secondary outcomes: Emotional function; Physical function; and Health-related quality of life.

Table S1. Search Strategy.

**CENTRAL (Cochrane Central Register of Controlled Trials)**
1. MeSH descriptor: [Aged] explode all trees
2. MeSH descriptor: [Aging] explode all trees
3. #1 OR #2 OR aged OR elder* OR elderly OR older*
4. MeSH descriptor: [Exercise] explode all trees
5. MeSH descriptor: [Exercise Therapy] explode all trees
6. #4 OR #5 OR Exercise* OR "physical training" OR "physical endurance" OR "exercise training" OR *″physical activity″* OR ″physical fitness″ OR ″rehabilitation″ OR ″physical therapy modalities″ OR ″exercise therapy″
7. MeSH descriptor: [Lung Neoplasms] explode all trees
8. #7 OR "lung carcinoma" OR "pulmonary carcinoma" OR "lung neoplasm" OR "lung neoplasms" OR "pulmonary neoplasm" OR "pulmonary neoplasms" OR "pulmonary cancer" OR "lung cancer" OR "lung tumor" OR "lung tumour" OR "lung metastases" OR OR "Non-Small-Cell Lung"
9. "general surgery" OR "pulmonary surgical procedures" OR "thoracic surgical procedures" OR "surgical procedures, operative" OR "surg*" OR "operat*" OR "resection"
10. #3 AND #6 AND #8 AND #9
11. Trials
12. Year first published 1970-2018
13. Date added to CENTRAL trials database: 01/01/1970 – 13/02/2018
CINAHL

1. ((MH "Aged+") OR "aged" OR (MH "Aging+") OR "elder*" OR "elderly" OR "older*")<br>2. AND (physical training OR (MH "Therapeutic Exercise+") OR (MH "Rehabilitation Exercise (Saba CCC)/EV/TH/UT") OR (MH "Exercise+/EV/MA/UT") OR (MH "Physical Activity (Omaha)/UT") OR ″physical fitness″ OR ″rehabilitation″ OR ″physical therapy modalities″ OR ″exercise therapy″*)*<br>3. AND ((MH "Lung Neoplasms+/RH/TH/PC") OR "Lung Neoplasms" OR "lung carcinoma" OR "pulmonary carcinoma" OR "lung neoplasm" OR "pulmonary neoplasm" OR "pulmonary neoplasms" OR "pulmonary cancer" OR "lung cancer" OR "lung tumor" OR "lung tumour" OR "lung metastases" OR "Non-Small-Cell Lung")<br>4. AND ("general surgery" OR "pulmonary surgical procedures" OR "thoracic surgical procedures" OR "surgical procedures, operative" OR "surg*" OR "operat*" OR "resection")<br>5. Language: english<br>6. Age: aged: 65+ years, middle aged: 45-64 years<br>7. Publication years: 1970-2018
EMBASE
1. ("lung neoplasms/exp" OR "lung carcinoma" OR "pulmonary carcinoma" OR "lung neoplasm" OR "lung neoplasms" OR "pulmonary neoplasm" OR "pulmonary neoplasms" OR "pulmonary cancer" OR "lung cancer" OR "lung tumor" OR "lung tumour" OR "lung metastases" OR "Non-Small-Cell Lung")<br>2. ("exercise therapy" OR exercise/exp OR exercise* OR "physical training" OR "physical endurance" OR "exercise training" OR *″physical activity″* OR ″physical fitness″ OR ″rehabilitation″ OR ″physical therapy modalities″ OR ″exercise therapy″*)*<br>3. (aged/exp OR aged OR aging OR elder* OR elderly OR older*)<br>4. ("general surgery" OR "pulmonary surgical procedures" OR "thoracic surgical procedures" OR "surgical procedures, operative" OR "surg*" OR "operat*" OR "resection")<br>5. (#1 AND #2 AND #3 AND #4)<br>6. Study type: human, controlled study<br>7. Publication type: Article<br>8. Publication year: 1970-2018<br>9. Age: aged: 65+ years, middle aged: 45-64 years
OVID
1. "lung neoplasms/exp" or "lung carcinoma" or "pulmonary carcinoma" or "lung neoplasm" or "lung neoplasms" or "pulmonary neoplasm" or "pulmonary neoplasms" or "pulmonary cancer" or "lung cancer" or "lung tumor" or "lung tumour" or "lung metastases" OR "Non-Small-Cell Lung").mp. [mp=ti, ot, ab, tx, kw, ct, sh, hw]<br>2. "exercise therapy".mp. or exercise/exp or exercise*.mp. or "physical training".mp. or "physical endurance".mp. or "exercise training".mp. or "physical activity".mp. or "physical fitness".mp. or "rehabilitation".mp. or "physical therapy modalities".mp. or "exercise therapy".mp. [mp=ti, ot, ab, tx, kw, ct, sh, hw]<br>3. aged/exp or aged.mp. or aging.mp. or elder*.mp. or elderly.mp. or older*.mp. [mp=ti, ot, ab, tx, kw, ct, sh, hw]<br>4. ("general surgery" or "pulmonary surgical procedures" or "thoracic surgical procedures" or "surgical procedures, operative" or "surg*" or "operat*" or "resection").mp. [mp=ti, ot, ab, tx, kw, ct, sh, hw]<br>5. 1 and 2 and 3 and 4<br>6. limit 7 to "middle aged (45 plus years)" [Limit not valid in CDSR,ACP Journal Club,DARE,CCA,CCTR,CLCMR; records were retained]<br>7. Publication year: 1970-2018<br>8. Additional limits: CheckTags (Humans), language: English, Publication Types: Controlled Clinical Trial
PUBMED
Search (aged[Mesh] OR aged[title] OR aging[Mesh] OR elder*[title] OR elderly OR older*) AND (exercise[Mesh] OR "Exercise therapy"[Mesh] OR Exercise*[title] OR "physical training" OR "physical endurance" OR "exercise training" OR *″physical activity″* OR ″physical fitness″ OR ″rehabilitation″ OR ″physical therapy modalities″ OR ″exercise therapy″) AND ("Lung Neoplasms"[Mesh] OR "lung carcinoma" OR "pulmonary carcinoma" [title] OR "lung neoplasm" OR "lung neoplasms" OR "pulmonary neoplasm" OR "pulmonary neoplasms" OR "pulmonary cancer" OR "lung cancer"[title] OR "lung tumor"[title] OR "lung tumour"[title] OR "lung metastases" OR "Non-Small-Cell Lung" AND "general surgery" OR "pulmonary surgical procedures" OR "thoracic surgical procedures" OR "surgical procedures, operative" OR "surg*" OR "operat*" OR "resection") Sort by: Author Filters: Clinical Trial; Publication date from 1970/01/01 to 2018/02/13; Humans

SCOPUS
((TITLE-ABS-KEY ((*"Lung Neoplasms"* OR *"lung carcinoma"* OR *"pulmonary carcinoma"* OR *"lung neoplasm"* OR *"lung neoplasms"* OR *"pulmonary neoplasm"* OR *"pulmonary neoplasms"* OR *"pulmonary cancer"* OR *"lung cancer"* OR *"lung tumor"* OR *"lung tumour"* OR *"lung metastases"* OR "Non-Small-Cell Lung"))) AND (TITLE-ABS-KEY ((*"Exercise therapy"* OR *exercise** OR *"physical training"* OR *"physical endurance"* OR *"exercise training"* OR *"physical activity"* OR "physical fitness" OR "rehabilitation" OR "physical therapy modalities" OR "exercise therapy")))) AND (TITLE-ABS-KEY ((*aged* OR *aging* OR *elder** OR *elderly* OR *older**)))) AND (TITLE-ABS-KEY (("general surgery" OR "pulmonary surgical procedures" OR "thoracic surgical procedures" OR "surgical procedures, operative" OR "surg*" OR "operat*" OR "resection")))) AND TITLE-ABS-KEY (*"randomized controlled trials"*) AND (PUBYEAR > *1970*) AND (PUBYEAR < *2018*) AND (LIMIT-TO (DOCTYPE, *"ar"*)) AND (LIMIT-TO (LANGUAGE, *"English"*))AND (LIMIT-TO (EXACTKEYWORD, *"Humans"*)).
WEB OF SCIENCE
1. (TI=("Lung Neoplasms" OR "lung carcinoma" OR "pulmonary carcinoma" OR "lung neoplasm" OR "lung neoplasms" OR "pulmonary neoplasm" OR "pulmonary neoplasms" OR "pulmonary cancer" OR "lung cancer" OR "lung tumor" OR "lung tumour" OR "lung metastases" OR "Non-Small-Cell Lung")) AND LANGUAGE:(English)<br>Indexes=SCI-EXPANDED, SSCI, A&HCI, CPCI-S, CPCI-SSH, BKCI-S, BKCI-SSH, ESCI, CCR-EXPANDED, IC Timespan=All years<br>2. (TI=("Exercise therapy" OR Exercise* OR "physical training" OR "physical endurance" OR "exercise training" OR "physical activity" OR "physical fitness" OR "rehabilitation" OR "physical therapy modalities" OR "exercise therapy")) AND LANGUAGE: (English)<br>Indexes=SCI-EXPANDED, SSCI, A&HCI, CPCI-S, CPCI-SSH, BKCI-S, BKCI-SSH, ESCI, CCR-EXPANDED, IC Timespan=All years<br>3. (TI=(aged OR aging OR elder* OR elderly OR older*)) AND LANGUAGE: (English)<br>Indexes=SCI-EXPANDED, SSCI, A&HCI, CPCI-S, CPCI-SSH, BKCI-S, BKCI-SSH, ESCI, CCR-EXPANDED, IC Timespan=All years<br>4. (TI=("general surgery" OR "pulmonary surgical procedures" OR "thoracic surgical procedures" OR "surgical procedures, operative" OR "surg*" OR "operat*" OR "resection")) AND LANGUAGE: (English)<br>Indexes=SCI-EXPANDED, SSCI, A&HCI, CPCI-S, CPCI-SSH, BKCI-S, BKCI-SSH, ESCI, CCR-EXPANDED, IC Timespan=All years<br>5. (#1 AND #2 AND #3 AND #4) AND LANGUAGE: (English) AND DOCUMENT TYPES: (Article)<br>Indexes=SCI-EXPANDED, SSCI, A&HCI, CPCI-S, CPCI-SSH, BKCI-S, BKCI-SSH, ESCI, CCR-EXPANDED, IC Timespan=1970-2018

Table S2. Exclusion criteria and the list of excluded articles.

References	Reasons
[1]	There was no control group
[2]	Not a RCT
[3]	Not a RCT
[4]	Not a RCT
[5]	Not a RCT
[6]	Not a RCT
[7]	Not a RCT
[8]	Training in survivors
[9]	Training in survivors
[10]	Postoperative training
[11]	Postoperative training
[12]	Postoperative training
[13]	Postoperative training
[14]	Postoperative training
[15]	Postoperative training
[16]	Cancer training
[17]	Cancer training
[18]	Cancer training
[19]	Cancer training
[20]	Cancer training

[21]	Cancer training
[22]	Cancer training
[23]	Cancer training
[24]	Perioperative training
[25]	Perioperative training
[26]	Study other variables

References

1. Dimeo, F.C.; Thomas, F.; Raabe-Menssen, C.; Pröpper, F.; Mathias, M. Effect of aerobic exercise and relaxation training on fatigue and physical performance of cancer patients after surgery. A randomised controlled trial. Supportive Care in Cancer 2004, 12, 774–779, doi:10.1007/s00520-004-0676-4.
2. Andersen, A.H.; Vinther, A.; Poulsen, L.L.; Mellemgaard, A. Do patients with lung cancer benefit from physical exercise? Acta Oncol 2011, 50, 307–313, doi:10.3109/0284186x.2010.529461.
3. Bradley, A.; Marshall, A.; Stonehewer, L.; Reaper, L.; Parker, K.; Bevan-Smith, E.; Jordan, C.; Gillies, J.; Agostini, P.; Bishay, E., et al. Pulmonary rehabilitation programme for patients undergoing curative lung cancer surgery. Eur J Cardiothorac Surg 2013, 44, e266-271, doi:10.1093/ejcts/ezt381.
4. Hanna, L.R.; Avila, P.F.; Meteer, J.D.; Nicholas, D.R.; Kaminsky, L.A. The effects of a comprehensive exercise program on physical function, fatigue, and mood in patients with various types of cancer. Oncology Nursing Forum 2008, 35, 461–469, doi:10.1188/08.ONF.461-469.
5. Jones, L.W.; Peddle, C.J.; Eves, N.D.; Haykowsky, M.J.; Courneya, K.S.; Mackey, J.R.; Joy, A.A.; Kumar, V.; Winton, T.W.; Reiman, T. Effects of presurgical exercise training on cardiorespiratory fitness among patients undergoing thoracic surgery for malignant lung lesions. Cancer 2007, 110, 590–598, doi:10.1002/cncr.22830.
6. Naito, T.; Okayama, T.; Aoyama, T.; Mori, K.; Tanuma, A.; Takahashi, T. The role of exercise intervention in the management of cancer cachexia. Annals of Oncology 2015, 26, vii46, doi:10.1093/annonc/mdv439.3.
7. Quist, M.; Adamsen, L.; Rørth, M.; Laursen, J.H.; Christensen, K.B.; Langer, S.W. The Impact of a Multidimensional Exercise Intervention on Physical and Functional Capacity, Anxiety, and Depression in Patients with Advanced-Stage Lung Cancer Undergoing Chemotherapy. Integrative Cancer Therapies 2015, 14, 341–349, doi:10.1177/1534735415572887.
8. Litterini, A.J.; Fieler, V.K. The change in fatigue, strength, and quality of life following a physical therapist prescribed exercise program for cancer survivors. Rehabilitation Oncology 2008, 26, 11–17.
9. Repka, C.; Peterson, B.; Brown, J.; Lalonde, T.; Schneider, C.; Hayward, R. Cancer type does not affect exercise-mediated improvements in cardiorespiratory function and fatigue. In Integrative cancer therapies, 2014; Vol. 13, pp 473-481.
10. Brocki, B.; Andreasen, J.; Nielsen, L.; Nekrasas, V.; Gorst-Rasmussen, A.; Westerdahl, E. Short and long-term effects of supervised versus unsupervised exercise training on health-related quality of life and functional outcomes following lung cancer surgery - a randomized controlled trial. In Lung cancer (amsterdam, netherlands), 2014; Vol. 83, pp 102-108.
11. Arbane, G.; Tropman, D.; Jackson, D.; Garrod, R. Evaluation of an early exercise intervention after thoracotomy for non-small cell lung cancer (NSCLC), effects on quality of life, muscle strength and exercise tolerance: Randomised controlled trial. Lung Cancer 2011, 71, 229–234, doi:10.1016/j.lungcan.2010.04.025.
12. Arbane, G.; Douiri, A.; Hart, N.; Hopkinson, N.S.; Singh, S.; Speed, C.; Valladares, B.; Garrod, R. Effect of postoperative physical training on activity after curative surgery for non-small cell lung cancer: a multicentre randomised controlled trial. Physiotherapy 2014, 100, 100–107, doi:10.1016/j.physio.2013.12.002.
13. Edvardsen, E.; Skjønsberg, O.H.; Holme, I.; Nordsletten, L.; Borchsenius, F.; Anderssen, S.A. High-intensity training following lung cancer surgery: A randomised controlled trial. Thorax 2015, 70, 244–250, doi:10.1136/thoraxjnl-2014-205944.
14. Hoffman, A.J.; Brintnall, R.A.; Brown, J.K.; Eye, A.; Jones, L.W.; Alderink, G.; Ritz-Holland, D.; Enter, M.; Patzelt, L.H.; Vanotteren, G.M. Too sick not to exercise: using a 6-week, home-based exercise intervention for cancer-related fatigue self-management for postsurgical non-small cell lung cancer patients. Cancer Nurs 2013, 36, 175–188, doi:10.1097/NCC.0b013e31826c7763.

15. Stigt, J.; Uil, S.; Riesen, S.; Simons, F.; Denekamp, M.; Shahin, G.; Groen, H. A randomized controlled trial of postthoracotomy pulmonary rehabilitation in patients with resectable lung cancer. In Journal of thoracic oncology, 2013; Vol. 8, pp 214-221.
16. Chen, H.; Tsai, C.; Wu, Y.; Lin, K.; Lin, C. Randomised controlled trial on the effectiveness of home-based walking exercise on anxiety, depression and cancer-related symptoms in patients with lung cancer. In British journal of cancer, 2015; Vol. 112, pp 438-445.
17. Chen, H.; Tsai, C.; Wu, Y.; Lin, K.; Lin, C. Effect of walking on circadian rhythms and sleep quality of patients with lung cancer: a randomised controlled trial. In British journal of cancer, 2016; Vol. 115, pp 1304-1312.
18. Dhillon, H.; Bell, M.; Ploeg, H.; Turner, J.; Kabourakis, M.; Spencer, L.; Lewis, C.; Hui, R.; Blinman, P.; Clarke, S., et al. Impact of physical activity on fatigue and quality of life in people with advanced lung cancer: a randomized controlled trial. In Annals of oncology, 2017; Vol. 28, pp 1889-1897.
19. Henke, C.; Cabri, J.; Fricke, L.; Pankow, W.; Kandilakis, G.; Feyer, P.; Wit, M. Strength and endurance training in the treatment of lung cancer patients in stages IIIA/IIIB/IV. In Supportive care in cancer, 2014; Vol. 22, pp 95-101.
20. Oldervoll, L.M.; Loge, J.H.; Lydersen, S.; Paltiel, H.; Asp, M.B.; Nygaard, U.V.; Oredalen, E.; Frantzen, T.L.; Lesteberg, I.; Amundsen, L., et al. Physical exercise for cancer patients with advanced disease: A randomized controlled trial. Oncologist 2011, 16, 1649–1657, doi:10.1634/theoncologist.2011-0133.
21. Wiskemann, J.; Hummler, S.; Diepold, C.; Keil, M.; Abel, U.; Steindorf, K.; Beckhove, P.; Ulrich, C.M.; Steins, M.; Thomas, M. POSITIVE study: physical exercise program in non-operable lung cancer patients undergoing palliative treatment. BMC Cancer 2016, 16, 499, doi:10.1186/s12885-016-2561-1.
22. Hwang, C.; Yu, C.; Shih, J.; Yang, P.; Wu, Y. Effects of exercise training on exercise capacity in patients with non-small cell lung cancer receiving targeted therapy. In Supportive care in cancer, 2012; Vol. 20, pp 3169-3177.
23. Salhi, B.; Haenebalcke, C.; Perez-Bogerd, S.; Nguyen, M.D.; Ninane, V.; Malfait, T.L.A.; Vermaelen, K.Y.; Surmont, V.F.; Van Maele, G.; Colman, R., et al. Rehabilitation in patients with radically treated respiratory cancer: A randomised controlled trial comparing two training modalities. Lung Cancer 2015, 89, 167–174, doi:10.1016/j.lungcan.2015.05.013.
24. Sommer, M.; Trier, K.; Vibe-Petersen, J.; Missel, M.; Christensen, M.; Larsen, K.; Langer, S.; Hendriksen, C.; Clementsen, P.; Pedersen, J., et al. Perioperative rehabilitation in operation for lung cancer (PROLUCA) - rationale and design. In BMC cancer, 2014; Vol. 14, p 404.
25. Pehlivan, E.; Turna, A.; Gurses, A.; Gurses, H.N. The effects of preoperative short-term intense physical therapy in lung cancer patients: a randomized controlled trial. Ann Thorac Cardiovasc Surg 2011, 17, 461–468.
26. Wall, L. Changes in hope and power in lung cancer patients who exercise. In Nursing science quarterly, 2000; Vol. 13, pp 234–242.

© 2019 by the authors. Licensee MDPI, Basel, Switzerland. This article is an open access article distributed under the terms and conditions of the Creative Commons Attribution (CC BY) license (http://creativecommons.org/licenses/by/4.0/).
